# Systemic Lupus Erythematosus Presenting with Ischemic Proctitis and Abdominal Compartment Syndrome

**DOI:** 10.1155/2020/5723403

**Published:** 2020-02-13

**Authors:** Yousaf Bashir Hadi, John Lindsay, Syeda Fatima Zehra Naqvi, Hatim Al-Jaroushi

**Affiliations:** ^1^Department of Medicine, West Virginia University, Morgantown, WV, USA; ^2^West Virginia University School of Medicine, Morgantown, WV, USA; ^3^Department of Medicine, Section of Pulmonary and Critical Care Medicine, West Virginia University, Morgantown, WV, USA

## Abstract

Ischemic colitis and proctitis is a rare manifestation of systemic lupus erythematosus (SLE) and results from mesenteric vasculitis. Owing to diverse blood supply and presence of multiple collaterals, rectum is the least effected site in SLE enteritis. Ischemic proctocolitis as the presenting feature of SLE is exceedingly rare, with only three cases reported in the published scientific literature. We present the first case of SLE presenting as ischemic proctitis, leading to intraperitoneal hemorrhage and abdominal compartment syndrome. A young lady presented with ischemic proctitis and a hematoma masquerading as a pelvic mass, with subsequent development of massive intraperitoneal hemorrhage, shock, and rectal perforation. The patient required urgent surgery and was initiated on high-dose steroids.

## 1. Introduction

Ischemic colitis and proctitis is a rare manifestation of systemic lupus erythematosus (SLE). It results from small-vessel vasculitis and is accompanied by high SLE disease activity, and can affect the small or large intestine. Diagnosis is confirmed with sigmoidoscopy and histopathology. Ischemic proctocolitis as the presenting feature of SLE is exceedingly rare, with only three cases reported in the published scientific literature [1–3]. No prior reports of hemorrhagic shock and perforations in this setting exist in the current literature. We present the first reported case of ischemic proctocolitis leading to massive intraperitoneal hemorrhage and hemorrhagic shock, with subsequent perforation as the initial presentation of SLE.

## 2. Case Presentation

A 39-year-old female presented to West Virginia University Hospitals' Intensive care unit as a transfer from an outside facility. She had initially presented to the outside facility with abdominal pain of 2 weeks' duration. The patient experienced worsening abdominal pain and weakness on the day of presentation, which prompted her to report to the outside facility. She was found to have a skin rash, involving her arms and face, which was biopsied, revealing a leukocytoclastic vasculitis. She was also found to have severe anemia and ascites. A computed tomography (CT) scan without contrast was concerning for an irregularity in the rectal lumen with a perirectal pelvic mass or hematoma. Over the course of her stay there, she developed hypotension and shock; she was intubated and transferred to our facility for further evaluation and management.

**Figure 1:**
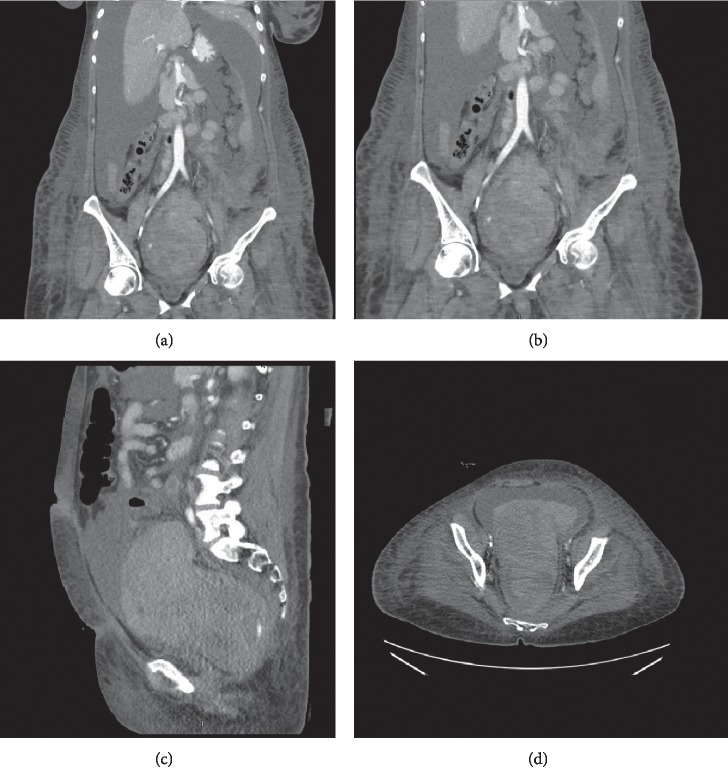
a, b) Coronal views of CT abdomen and pelvis with contrast show a heterogeneous, mass-like appearance of an organized rectal hematoma. (c) Sagittal view shows prominent rectal dilatation with high intensity signal showing probable active contrast extravasation. (d) Axial view of the hematoma.

On presentation to West Virginia University, she was found to be in shock. She endorsed a past history of thrombocytopenia; the patient had been told that she had idiopathic thrombocytopenic purpura and splenectomy. She reported lower abdominal pain. Her initial blood pressure was 82/50, and she was pale on examination with large, tense ascites. Her hemoglobin on arrival was 4.4 gram/dl, white blood cell count was 8500, and she had a fever of 101 degrees Fahrenheit. She was volume resuscitated with crystalloids and blood products, broad spectrum antibiotics were administered, and due to a concern for intra-abdominal bleeding, she was rushed to CT scanning. A CT scan of the abdomen and pelvis with contrast was obtained that revealed prominent distention of the rectum with heterogeneous density material within. It could represent a hematoma or a mass originating from a pelvic organ or the rectum itself. Large volume ascites was also noted, which likely represented hemorrhagic ascites. These findings can be seen on Figures 1(a)–1(d).

After returning from the scanner, the patient's hemodynamic state worsened; norepinephrine, vasopressin, and then phenylephrine were serially added subsequently. Her hemoglobin count was monitored closely, and she continued to require blood transfusions.

**Figure 2:**
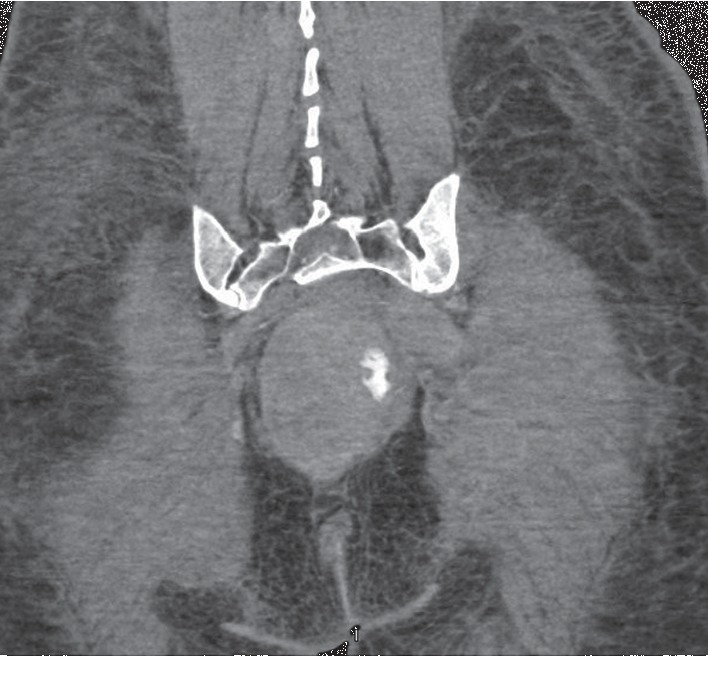
Sagittal view of CT angiogram showing active extravasation of contrast in the territory of the left internal iliac artery and inferior rectal artery.

After 10 hours of presentation, the patient had received 7 blood transfusions, and she remained in hemorrhagic shock. Her abdominal pressures continued to worsen, and the patient was developing abdominal compartment syndrome; she had developed anuria and renal failure with no urine output since presentation, and her respiratory status was worsening, requiring higher positive end expiratory pressures. General surgery service did not find her a candidate for exploratory laparotomy due to hesitation from the patient and her family and the unclear diagnosis at that time. A paracentesis was performed at the bedside, with removal of 4 liters of bloody fluid. The patient had considerable improvement in her hemodynamic and respiratory status after her paracentesis and was weaned off vasopressors. However, her bleeding failed to resolve, and she continued to require blood transfusions with redevelopment of hemorrhagic shock. A detailed discussion was held with the family, and the surgical service and surgery was deferred. The patient was sent for a CT angiogram after a second paracentesis and removal of 4 liters of blood from her peritoneal cavity. The CT angiogram revealed active contrast extravasation, and selective embolization of branches of her left inferior rectal and internal iliac vessel was performed.  Figure 2 shows the CT angiogram with active extravasation.

**Figure 3:**
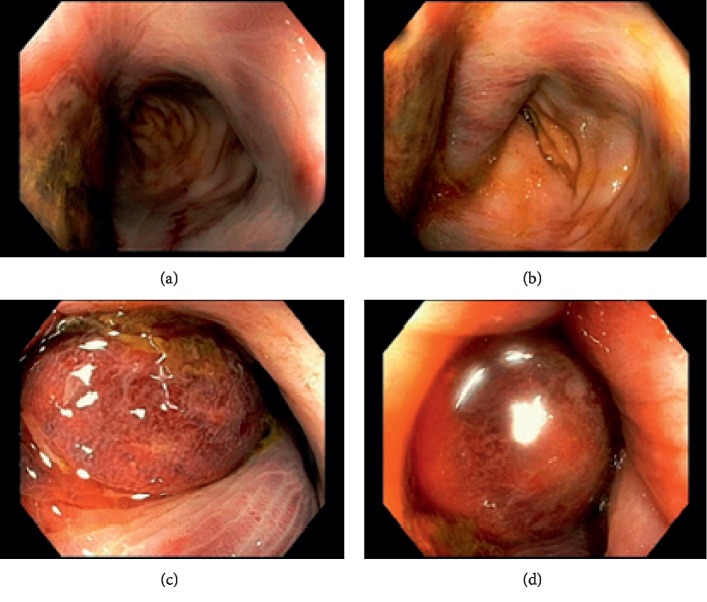
(a–d) Sigmoidoscopy images; an ischemic rectum with a bulging, tense hematoma in the rectal wall can be seen.

The patient received an urgent sigmoidoscopy on returning to her room from her angiogram that revealed an ischemic rectum with a bulging, tense hematoma in the rectal wall, and no associated mass was identified (Figure 3). Findings appeared consistent with ischemic proctitis. The patient's hemoglobin stabilized and her abdomen remained soft and nontender, with marked improvement in her respiratory and hemodynamic state after the embolization.

A serum vasculitis panel, antinuclear antibodies (ANA), Anti-double stranded DNA antibodies (Anti-DS DNA), anti-phospholipid antibodies, and complement levels were checked. However, the next day, the patient developed sudden abdominal pain and guarding, with abdominal exam consistent with peritonitis, and her hemodynamic state acutely worsened. A CT scan was obtained without contrast that revealed pneumoperitoneum out of proportion to the history of recent paracenteses, and she was emergently taken to the operating room for exploratory laparotomy and underwent proctectomy and colostomy with abdominal washout. A large amount of intraperitoneal clots were removed. The next day, the patient's ANA and DsDNA antibodies came back as positive at levels of 1 : 320 and 734 IU/dl (normal: <150 IU/dl), respectively. Low complement levels were found; C4 was 2 mg/dl (normal: 12–39 mg/dl), and C3 was 14 mg/dl (normal: 81–157 mg/dl). Anti-myeloperoxidase antibodies and phospholipid antibodies were negative, and anti-SS-A and SS-B antibodies also returned negative. A transthoracic echocardiogram revealed a mild-to-moderate pericardial effusion. The patient's biopsy showed two gross transmural perforations, ischemic proctocolitis, with acute serositis, and fat necrosis. Lymphocytic infiltration was noted. The patient was definitively diagnosed with ishemic proctocolitis secondary to active systemic lupus erythematosus, leading to intraperitoneal hemorrhage.

Over the course of the next three weeks, the patient was initiated on pulse dose methylprednisolone. The patient's renal function recovered. Rheumatology service was consulted, and the patient was initiated on hydroxychloroquine and was discharged after 3 weeks of surgery. At a follow-up of 6 months, the patient is doing well and continues her treatment for SLE. She has now been diagnosed with lupus nephritis with a renal biopsy.

## 3. Discussion

Gastrointestinal tract manifestations of SLE include intestinal pseudo-obstruction, lupus mesenteric vasculitis (LMV), protein losing enteropathy, or thrombosis of vasculature [4, 5]. Small bowel, usually the jejunum or ileum, seems to be most commonly affected [6]. Rarely, gastric and rectal involvement has also been described in the literature. Owing to diverse blood supply and presence of multiple collaterals, rectum is the least effected site in SLE enteritis. Previously, there are a total of three reports of proctitis as the presenting manifestation of SLE [1–3].

Lupus mesenteric vasculitis is the likely underlying mechanism of gut involvement in most of these cases. Unfortunately, no established histopathological hallmark of SLE enteritis currently exists [7]. Macroscopically and endoscopically, the appearance usually reflects edema, ulceration, gangrene, and rarely, perforation. GI involvement in SLE is usually diagnosed clinically, with imaging evidence as an adjunct, after exclusion of other gastrointestinal insults. Although SLE enteritis, with its reported prevalence of 0.2 to 9.7%, [8] is highly suspected in patients with established SLE, diagnosing SLE enteritis is a big challenge in patients with no previous SLE diagnosis.

Ischemic colitis is rare in SLE, with a reported incidence of 0.2%. However, it can prove life-threatening in cases where there is a delay in diagnosis and management [9, 10]. Our case resulted in a perforation and required emergent surgery, with significant associated morbidity. A high index of suspicion for SLE in patients presenting with fever and rash in an absence of infectious milieu can help expedite diagnosis and earlier initiation of immunosuppression. Our patient had initially presented with abdominal pain without ascites to the outside facility and developed shock and hemorrhage after a few days of presentation.

Treatment of SLE with vasculitis usually includes cyclophosphamide or some other immunosuppressant agent in addition to steroids. Our patient was initiated on methylprednisolone and hydroxychloroquine only. As the patient already showed a remarkable recovery, the addition of another agent was deferred. This level of response to steroids only is unusual.

Our patient was appropriately diagnosed with SLE based on serologic evidence; lack of probable alternate diagnoses, and fulfillment of the both the American College of Rheumatology (ACR) criteria and the Systemic Lupus International Collaborating Clinic (SLICC) criteria [11]. Initiation of steroids led to rapid recovery. Our patient had photosensitivity, rash, vasculitis, fever, and pericardial effusion, as well as positive serology. Although she self-reported being diagnosed with ITP in the past, serology from that time were not available.

In conclusion, we present an exceedingly rare presentation of SLE hitherto unreported in the published literature. Our patient presented with ischemic proctitis and a hematoma masquerading as a pelvic mass, with subsequent development of massive intraperitoneal hemorrhage, shock, and rectal perforation, as the presenting manifestation of SLE.
